# Mining Database to Identify Aging-Related Molecular Subtype and Prognostic Signature in Lung Adenocarcinoma

**DOI:** 10.1155/2022/9142903

**Published:** 2022-10-11

**Authors:** Caihou Feng, Weibi Che, Hanping Liang, Hai Zhang, Cong Lan, Bomeng Wu, Wanli Lin, Ying Chen

**Affiliations:** Department of Thoracic Surgery, Gaozhou People's Hospital, Gaozhou 525200, China

## Abstract

**Background:**

Lung cancer is emerging as one of most deadly diseases, and the mortality rate was still high with 5-year overall survival rate less than 20%. Aging is referred as protumorigenic state, and it plays a significant role in cancer development.

**Methods:**

Molecular subtype of lung cancer was identified by consensus cluster analysis. Prognostic signature was constructed using LASSO cox regression analysis. CeRNA network was constructed to explore lncRNA-miRNA-mRNA regulatory axis.

**Results:**

A total of 27 differentially expressed aging-related genes (ARGs) were obtained in LUAD. Three clusters of TCGA-LUAD patients with significant difference in prognosis, immune infiltration, chemotherapy, and targeted therapy were identified. We also developed an aging-related prognostic signature that had a better performance in predicting the1-year, 3-year, and 5-year overall survival of LUAD. Further analysis suggested a significant correlation between prognostic signature gene expression and clinical stage, immune infiltration, tumor mutation burden, microsatellite instability, and drug sensitivity. We also identified the lncRNA UCA1/miR-143-3p/CDK1 regulatory axis in LUAD.

**Conclusion:**

Our study identified three clusters of TCGA-LUAD patients with significant difference in prognosis, immune infiltration, chemotherapy, and targeted therapy. We also developed an aging-related prognostic signature that had a good performance in the prognosis of LUAD.

## 1. Introduction

Lung cancer is emerging as one of most deadly diseases with an estimated 2.09 million new cases and 1.76 million deaths per year globally [1]. Lung adenocarcinoma (LUAD) is the main type of lung cancer. Despite great advance had been achieved in the diagnosis and therapy of lung cancer, the mortality rate was still high, and 5-year overall survival rate is less than 20% [2, 3]. Apart from the TNM staging system, there are still no ideal biomarkers or signatures to predict the prognosis of lung cancer patients. Increasing evidences revealed that molecularly-defined subtypes could provide novel strategies for the therapy and prognosis of lung cancer [4]. Thus, it is significance to develop effective prognostic signature and molecular subtype for lung cancer.

Aging is a complex process associated with various molecular and cellular mechanisms [5]. Aging facilitates a series of degenerative pathologies with characteristics of debilitating losses of tissue or cellular function [6]. The incidence of malignancy increases as the age increases [7]. Moreover, aging is referred as protumorigenic state, and it plays a significant role in cancer development [8]. Moreover, aging-related signature could serve as prognostic biomarker for many types of cancers, including colorectal cancer [9], ovarian cancer [10], and glioma [11]. However, the significance role of aging-related genes (ARGs) in LUAD had not been clarified.

In the current study, we conducted consensus clustering for differently expressed ARGs in the Cancer Genome Atlas (TCGA) database. This was followed by the correlation analysis between molecular subtype and drug sensitivity as well as immune infiltration. We then developed a prognostic risk model based on prognostic ARGs. Moreover, we also clarified the potential molecular mechanism by constructing a ceRNA network. Our result may reveal the potential implication of ARGs as marker for the prognosis and therapy of LUAD patients.

## 2. Materials and Methods

### 2.1. Data Acquisition and Preprocessing

Human ARGs were isolated from HAGR on March 1, 2022 (*n* = 307, http://genomics.senescence.info/genes/, Supplementary Table 1) [12]. Gene expression profile of LUAD was isolated from the TCGA database (https://portal.gdc.cancer.gov/), using the limma package in the *R* software to study the differentially expressed genes (DEGs) with adjusted *P* < 0.05 and fold change > 2 as the threshold value. The differentially expressed ARGs were shown with Venn diagram. The somatic mutation data of LUAD was downloaded from UCSC Xena (https://xena.ucsc.edu/), and the result was shown with “maftools” package. Copy number variation (CNV) oncoplot of differentially expressed ARGs in LUAD was drawn with GISTIC2.0 [13].

### 2.2. Consensus Cluster Analysis

Based on differentially expressed ARGs, we then identified the optimum number of clusters of LUAD with “ConsensusClusterPlus” package. The survival curve of each cluster in LUAD was drawn using “survival” package. Moreover, immuneeconv algorithm were used to evaluate the immune score. The immune cell abundance and immune-checkpoint-related gene expression in each cluster was evaluated with Student *t*-test with “ggplot 2” package. Moreover, we also used “pRRophetic” *R* package to calculate the chemotherapeutic response in each cluster.

### 2.3. Development of Aging-Related Prognostic Signature Analysis

Univariate cox proportional hazard regression analysis was conducted to explore aging-related prognostic genes (*P* < 0.05). This was followed by the development of aging-related prognostic signature by using the least absolute shrinkage and selection operator (LASSO) regression algorithm in “glmnet” package. The risk score of LUAD cases was established as follows: risk score = ∑*i* = 1nCoef(*i*) × x(*i*). The nearest neighbor estimation (NNE) method was utilized to evaluate the 3-year survival and 5-year survival of LUAD. ROC curve was drawn with “survivalROC” *R* package. Using “ggDCA” package, we also draw a decision curve analysis (DCA) to evaluate the prediction ability of this signature. Further, Pearson correlation analysis was conducted to analyze the correlation between risk score and immune infiltration.

### 2.4. Risk Module Gene Analysis

Kruskal-Wallis test was performed to evaluate the differences of the risk module gene expression in different stages of LUAD patients. After we obtained the TMB/MSI score of LUAD patients from the TCGA database, we then analyzed their correlation with the risk module gene expression with Spearman's method. Pearson correlation analysis was performed to get the correlation between risk module gene expression and drug IC50 of Genomics of Therapeutics Response Portal (CTRP) and immune cell abundance of TIMER (https://cistrome.shinyapps.io/timer/). After we downloaded the cell line mRNA expression profile from the CCLE dataset (https://portals.broadinstitute.org/ccle), we then explored the risk module gene expression in different types of LUAD cell with “ggplot 2” package. The miRNA targets were identified by using miRDB (http://mirdb.org/), StarBase (http://starbase.sysu.edu.cn/), and miRWalk (http://mirwalk.umm.uni-heidelberg.de/). And the lncRNA targets were identified by LncBase (http://carolina.imis.athena-innovation.gr/) and StarBase (http://starbase.sysu.edu.cn/).

## 3. Results

### 3.1. Identification of ARG Expression and Their Mutation Landscape of in LUAD

**Figure 1:**
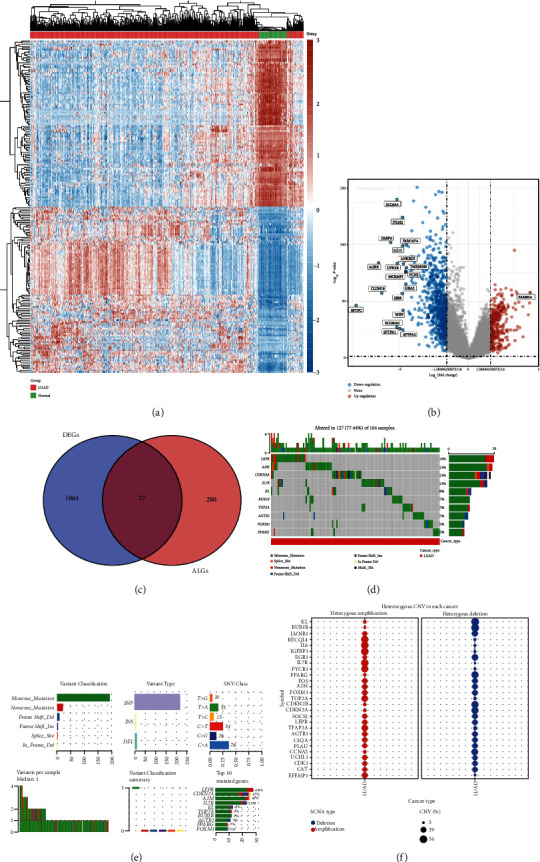
Expression and mutation landscape of ARGs in LUAD. (a, b) The volcano plot and heat map revealed the differentially expressed genes in LUAD. (c) Venn diagram revealed the differentially expressed ARGs in LUAD. (d, e) The SNV landscape of ARGs in LUAD. (d) The CNV landscape of ARGs in LAUD. ARG: aging-related gene subtype; DEGs: differentially expressed genes.

Figures 1(a) and 1(b) show the DEGs in LUAD. And a total of 1091 DEGs were identified. Among these DEGs, 27 was differentially expressed ARGs (Figure 1(c)). We then explored the genetic mutation of these 27 differentially expressed ARGs in LUAD, and the results were shown in Figures 1(d)–1(f). The data revealed that top 3 genes with the highest mutation frequency in LUAD were LEPR, A2M, and CDKN2A (Figures 1(d) and 1(e)). CNV analysis demonstrated a significant homozygous deletion of KL, BUB1B, and LMNB1 in LUAD (Figure 1(f)). Moreover, most differentially expressed ARGs had a homozygous amplification in LUAD (Figure 1(f)).

### 3.2. Consensus Clustering of ARGs in Three Clusters in LUAD

**Figure 2:**
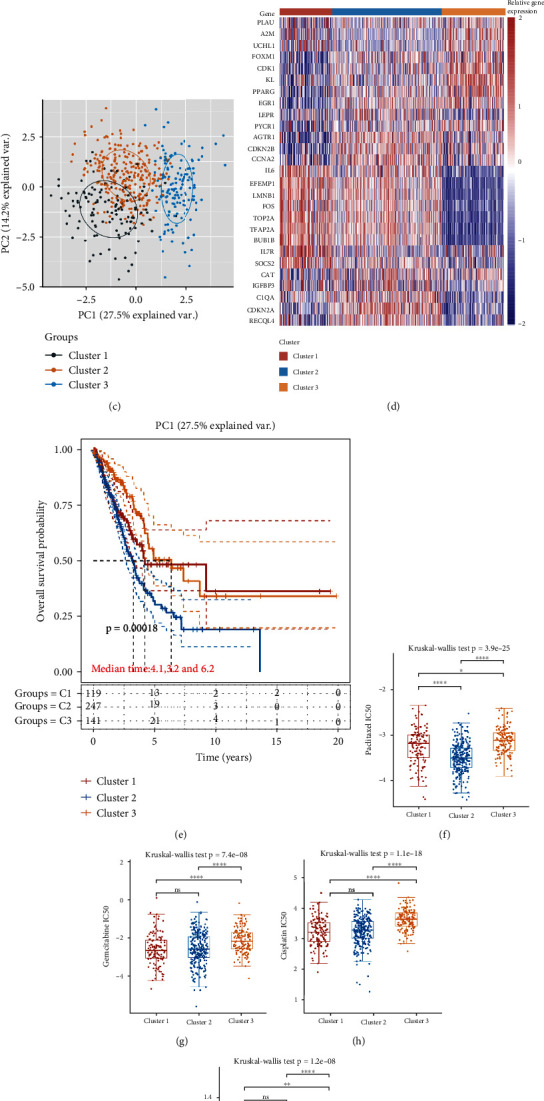
Consensus clustering of ARGs in LUAD. (a, b) Consensus clustering cumulative distribution function (CDF), relative change in area under CDF curve, and tracking plot for *k* = 2 − 6. (c, d) LUAD cases were divided into three clusters. (e) The overall survival curves in clusters 1/2/3 of LUAD cases. (f)–(i) The distribution of IC50 score of three clusters in LUAD. ^∗^*P* < 0.05, ^∗∗^*P* < 0.01, ^∗∗^*P* < 0.0001. ARG: aging-related gene subtype.

**Figure 3:**
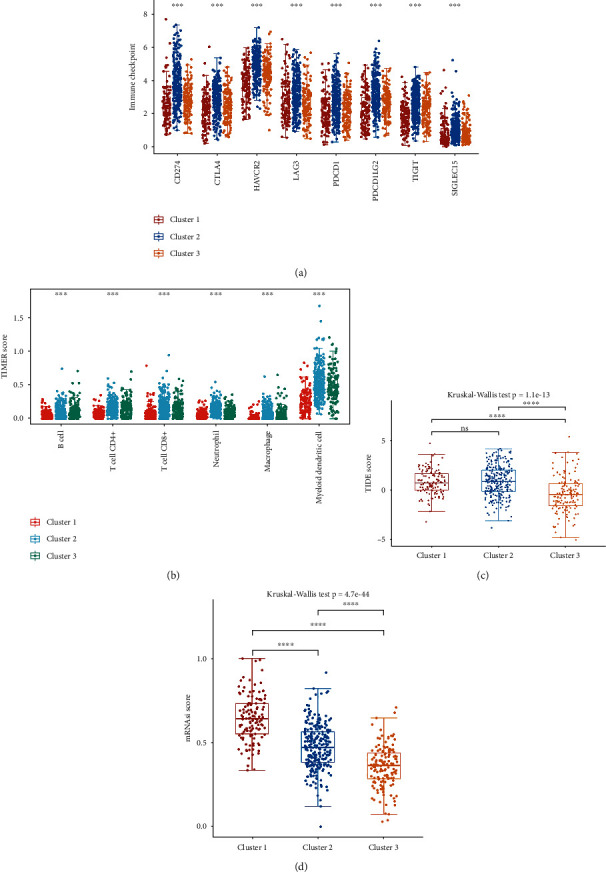
The difference of immune infiltration in three clusters of LUAD. (a) The differences of immune checkpoint gene expression of three clusters in LUAD. (b) The differences of TIMER score of three clusters in LUAD. (c, d) The differences of TIDE and mRNAsi score of three clusters in LUAD. ^∗∗∗^*P* < 0.001, ^∗∗∗∗^*P* < 0.0001.

Consensus clustering analysis was utilized to distinguish LUAD patients based on 27 differently expressed ARGs. Interestingly, these differently expressed ARGs could separate TCGA-LUAD patients into three categories according to CDF values and delta area (Figures 2(a)–2(d) ). Among these categories, LUAD patients in cluster 2 had a worse prognosis while LUAD patients in cluster 3 had a best prognosis (Figure 2(e), *p* = 0.00018). Considering the significant role of chemotherapy and targeted therapy in LUAD, we then evaluate the response of this three clusters to some common chemotherapeutic drugs and targeted therapeutics. The data suggested that LUAD patients in cluster 3 could be more resistant to commonly chemotherapy and targeted therapy, including paclitaxel, gemcitabine, cisplatin, and gefitinib (Figures 2(f)–2(i), all *P* < 0.05). Increasing evidences suggested immunotherapy as the most promising therapeutic strategy for LUAD patients in advance stage [14, 15]. In the current study, the data demonstrated an immune checkpoint expression (Figure 3(a), all *P* < 0.05), immune score (Figure 3(b), *p* < 0.05), and TIDE score (Figure 3(c), all *p* = 1.1*e* − 13) in cluster 2 versus cluster 1 and cluster 3 in LUAD. Cancer stem cells (CSCs) are believed to be responsible for tumor growth and maintenance, and they are involved in the resistance to conventional chemotherapy and radiation and tumor metastasis and recurrence. In our study, we found that LUAD patients in cluster 1 had a higher mRNAsi score versus cluster 2 and cluster 3 (Figure 3(d), *p* = 4.7*e* − 44).

### 3.3. The Functional Enrichment of ARGs

**Figure 4:**
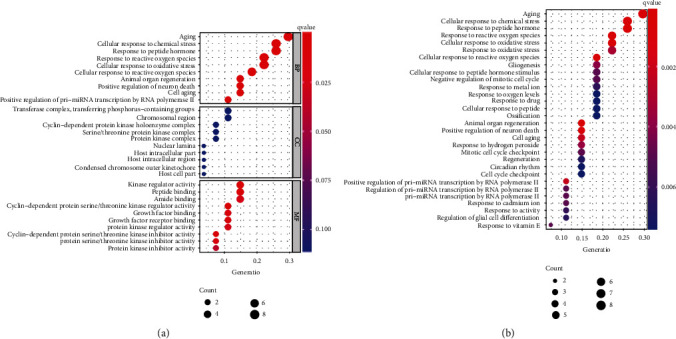
The enriched items in functional enrichment analysis. (a) The enriched items in gene ontology analysis. (b) The enriched items in Kyoto Encyclopedia of Genes and Genomes pathways analysis. BP: biological process; CC: cellular component; MF: molecular function.

The GO analysis revealed that these ARGs were mainly associated with aging, cellular response to oxidative stress, cell aging, positive regulation of pri-miRNA transcription by RNA polymerase II, kinase regulator activity, growth factor binding, and protein kinase inhibitor activity (Figure 4(a)). Moreover, ARGs were mainly associated with aging, cellular response to chemical stress, gliogenesis, response to drug, mitolic cell cycle checkpoint, and circadian rhythm in KEGG analysis (Figure 4(b)).

### 3.4. The Prognostic Significance of ARGs in LUAD

**Figure 5:**
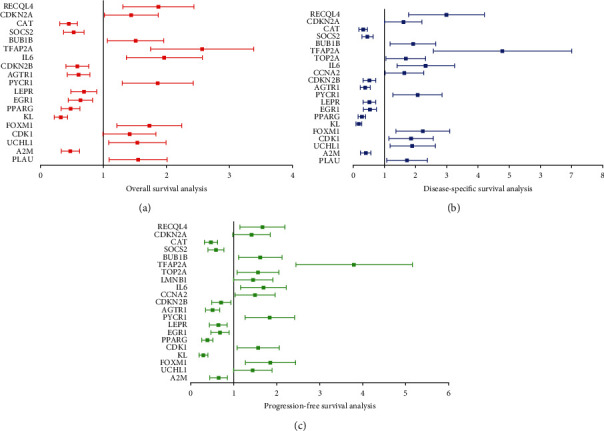
The result of prognosis of ARGs in LUAD. (a) Forest map revealed the result of overall survival of ARGs in LUAD. (b) Forest map revealed the result of disease-specific survival of ARGs in LUAD. (c) Forest map revealed the result of progression-free survival of ARGs in LUAD. ARG: aging-related gene.

We then evaluate the prognostic value of ARGs in LUAD. In overall survival analysis, a total of 19 ARGs (PLAU, A2M, UCHL1, FOXM1, CDK1, KL, PPARG, EGR1, LEPR, PYCR1, AGTR1, CDKN2B, IL6, TFAP2A, BUB1B, SOCS2, CAT, CDKN2A, and RECQL4) were correlated with the overall survival of LUAD patients (Figure 5(a), all *P* < 0.05). Moreover, a total of 21 ARGs (PLAU, A2M, UCHL1, FOXM1, CDK1, KL, PPARG, EGR1, LEPR, PYCR1, AGTR1, CDKN2B, CCNA2, IL6, TOP2A, TFAP2A, BUB1B, SOCS2, CAT, CDKN2A, and RECQL4) were correlated with the disease-specific survival of LUAD patients (Figure 5(b), all *P* < 0.05). As for progression-free survival, a total of ARGs (A2M, UCHL1, FOXM1, CDK1, KL, PPARG, EGR1, LEPR, PYCR1, AGTR1, CDKN2B, CCNA2, IL6, LMNB1, TOP2A, TFAP2A, BUB1B, SOCS2, CAT, CDKN2A, and RECQL4) were correlated with the prognosis of LUAD patients (Figure 5(c), all *P* < 0.05). Combined with these results, a total of 17 ARGs were suggested as potential prognostic biomarkers for LUAD, including AGTR1, BUB1B, CAT, CDK1, CDKN2A, CDKN2B, EGR1, FOXM1, IL6, KL, LEPR, PPARG, PYCR1, RECQL4, SOCS2, TFAP2A, and UCHL1.

### 3.5. Development of an Aging-Related Prognostic Signature for LUAD

**Figure 6:**
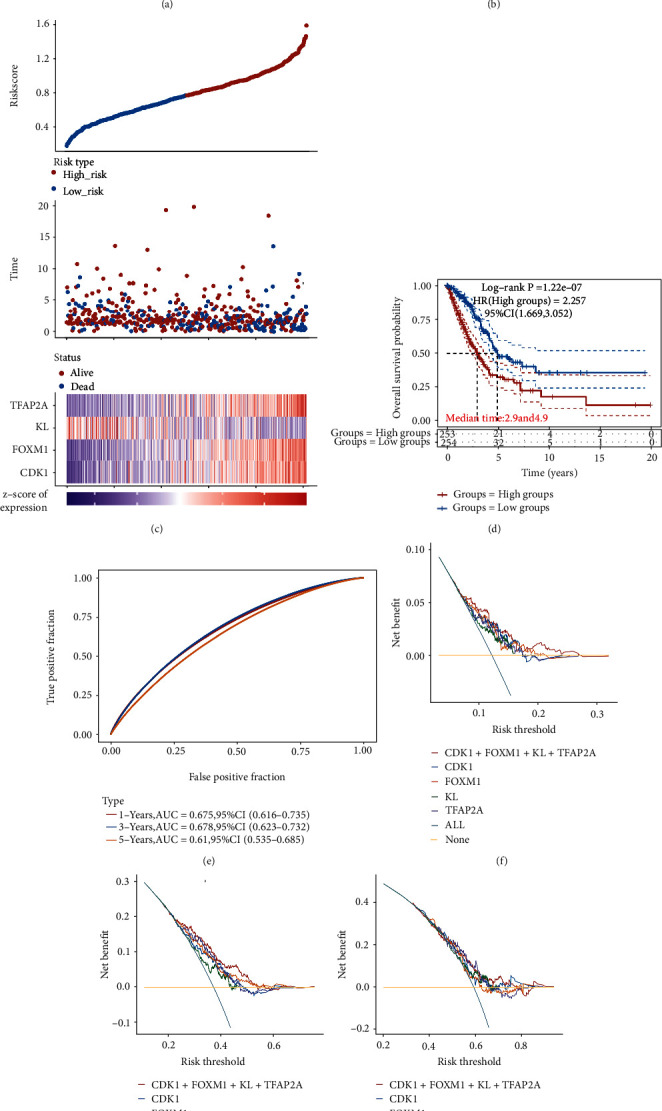
Aging-related prognostic signature in LUAD. (a, b) The coefficient and partial likelihood deviance of prognostic signature. (c) The risk score distribution, patient survival status, and aging-related gene expression profile of prognostic signature. (d) Overall survival curve of LUAD patients in the high-risk and low risk group. (e) ROC curve evaluated the predictive value of this prognostic signature in 1-year, 3-year, and 5-year overall survival. (f)–(h) Decision curve analysis of candidate prognostic biomarker or signature for predicting 1-year, 3-year, and 5-year survival status of LUAD patients.

**Figure 7:**
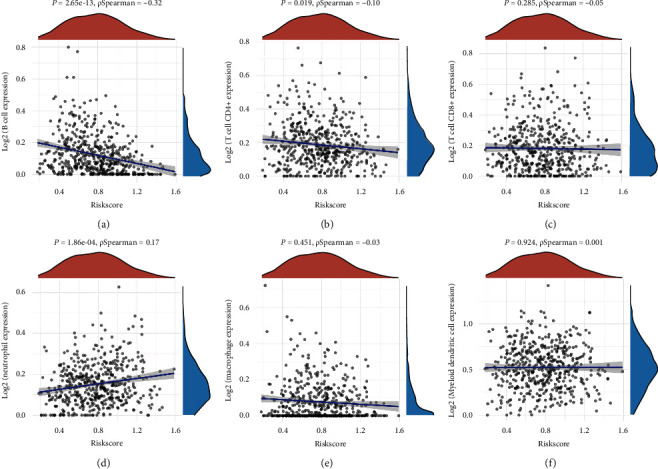
The correlation between risk score and immune infiltration in LUAD. The correlation between risk score and the abundance of B cells (a), CD4+ T cells (b), CD8+ T cells (c), Neutrophils (d), macrophage (e), and dendritic cells (f) in LUAD.

LASSO cox regression analysis was performed with 17 potential prognostic biomarkers to develop an aging-related prognostic signature for LUAD. As a result, four aging-related genes were included in this prognostic signature. The coefficient and partial likelihood deviance of prognostic signature was shown in Figures 6(a) and 6(b). The risk score, survival status, and gene expression of prognostic signature were shown in Figure 6(c). Overall survival analysis revealed that LUAD patients with high risks core had a poor overall survival (Figure 6(d), *p* = 1.22*e* − 7), and the AUC was 0.675, 0.678, and 0.61 in 1-year, 3-year, and 5-year, respectively, in ROC curve. Further decision curve analysis (DCA) revealed that this aging-related prognostic signature had a better performance in predicting the1-year, 3-year, and 5-year OS of LUAD patients versus potential aging-related prognostic biomarkers (Figures 6(f)–6(h)). We then analyzed the correlation between the risk score of LUAD patients and immune cell infiltration, which demonstrated a negative correlation between risk score and the abundance of B cells (Figure 7(a), *p* = 2.65*e* − 12) and CD4+ T cells (Figure 7(b), *p* = 0.01). Moreover, risk score showed positive correlation with neutrophil immune infiltration (Figure 7(d), *p* = 1.86*e* − 4). However, there is no significant correlation between risk score and the abundance of CD8+ T cells (Figure 7(c)), macrophage (Figure 7(e)), and dendritic cells (Figure 7(f)).

### 3.6. Comprehensive Analysis of Prognostic Signature Genes

**Figure 8:**
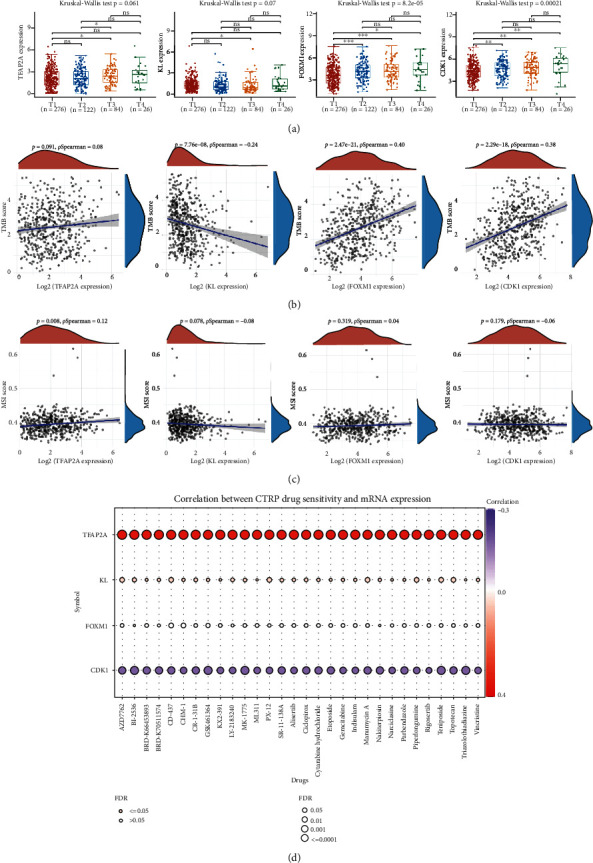
Comprehensive analysis of prognostic signature genes in LUAD. (a) The expression of TFAP2A, KL, FOXM1, and CDK1 in different clinical stages of LUAD. (b, c) Correlation between TMB/MSI score and prognostic signature genes expression in LUAD. (d) Correlation between drug sensitivity and prognostic signature genes expression in LUAD. ^∗^*P* < 0.05; ^∗∗^*P* < 0.01; ^∗∗∗^*P* < 0.001.

**Figure 9:**
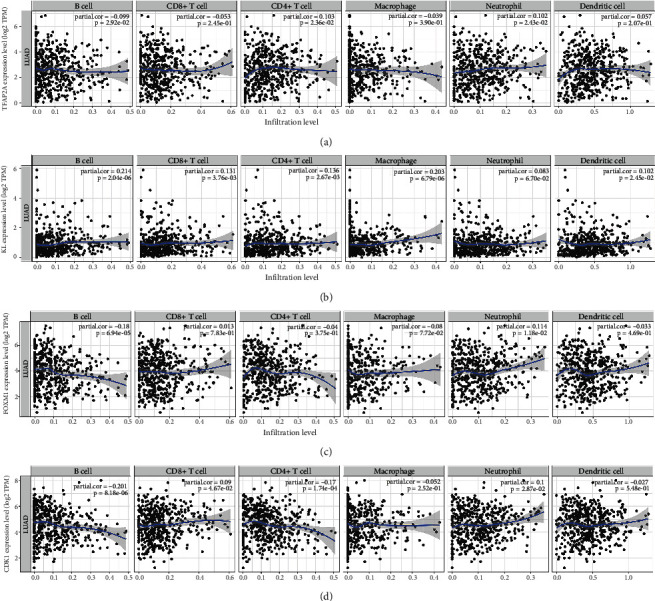
The correlation between prognostic signature genes and immune infiltration in LUAD. The correlation between the expression of TFAP2A (a), KL (b), FOXM1 (c), and CDK1 (d) and immune infiltration in LUAD.

The expression of FOXM1 (*p* = 8.2*e* − 5) and CDK1 (*p* = 0.00021) increases as clinical stage is increasing in LUAD (Figure 8(a)), suggested that FOXM1 and CDK1 may be involved in the progression of LUAD. TMB and MSI were suggested as predictive markers for tumor immunotherapy efficacy [16]. In our study, the TMB score showed negative association with KL expression (*p* = 7.76*e* − 8) and positive association with FOXM1 expression (*p* = 2.47*e* − 21) and CDK1 expression (*p* = 2.2*e* − 18) (Figure 8(b)). Moreover, the TFAP2A expression was positively correlated with MSI score in LUAD (Figure 8(c), *p* = 0.008). We then analyzed the correlation between the correlation between existing therapy target and gene expression. The current study revealed that high TFAP2A expression and low CDK1 expression could be more resistant to drug resistance in LUAD (Figure 8(d)). We also analyzed the correlation between prognostic signature genes expression and immune cell infiltration in LUAD. As a result, the TFAP2A expression showed positive correlation with the level of CD4+ T cells and neutrophils (Figure 9(a)). The KL expression increased as the abundance of B cells, CD4+ T cells, CD8+ T cells, macrophage, and dendritic cells increased (Figure 9(b)). Moreover, the FOXM1 expression was significantly correlated with B cell infiltration and neutrophils (Figure 9(c)). The CDK1 expression decreased as the abundance of B cells and CD4+ T cells increased (Figure 9(d)).

### 3.7. lncRNA-miRNA-mRNA Regulatory Axis Analysis

**Figure 10:**
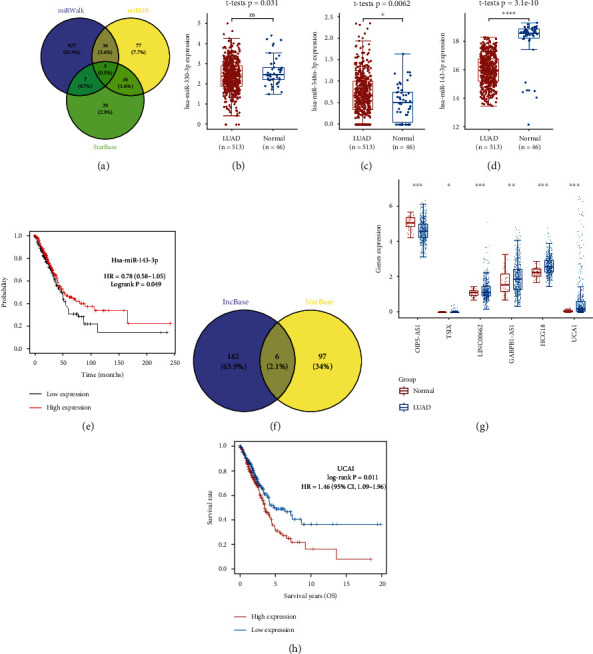
lncRNA-miRNA-mRNA regulatory axis in LUAD. (a) The miRNA targets of CDK1 predicted by miRDB, miRWalk and StarBase. (b)–(e) The expression and prognosis significance of miRNA targets in LUAD. (f) The lncRNA targets of miR-143-5p predicted by LncBase and StarBase. (g, h) The expression and prognosis significance of lncRNA targets in LUAD. ^∗^*P* < 0.05; ^∗∗^*P* < 0.01; ^∗∗∗^*P* < 0.001.

We selected CDK1 for further analysis. Finally, an lncRNA-miRNA-mRNA regulatory axis analysis was performed to further clarify potential mechanism. As a result, five miRNAs (miR-374c-3p, miR-143-3p, miR-330-3p, miR-641, and miR-548o-3p) were suggested as miRNA target of CDK1 based on the data of miRDB, miRWalk, and StarBase) (Figure 10(a)). Among these 5 miRNAs, 3 miRNAs (miR-143-3p, miR-330-3p, and miR-548o-3p) were differentially expressed in LUAD (Figures 10(b)–10(d)), and only 1 miRNA (miR-143-3p) was significantly associated with prognosis of LUAD (Figure 10(e)). Thus, miR-143-3p may be the most promising miRNA target of CDK1. We then explored the lncRNA target of miR-143-3p. As a result, a total of 6 lncRNAs (OIP5-AS1, TSIX, LINC00662, GABPB1-AS1, HCG18, UCA1) were suggested as the targets of miR-143-3p based on the data of StarBase and LncBase (Figure 10(f)). Though all these 6 lncRNAs were differentially expressed in LUAD (Figure 10(g)), only UCA1 was significantly correlated with the prognosis of LUAD (Figure 10(h)). Thus, it may be the most promising lncRNA target of miR-143-3p. All in all, the lncRNA UCA1/miR-143-3p/CDK1 regulatory axis may play a vital role in the progression in LUAD, and further in vivo and in vitro studies should be conducted to verify this hypothesis.

## 4. Discussion

As one of a vital risk factor for malignancies, aging exerts a vital role in human morbidity and mortality [17]. Aging-related genes were suggested as prognostic biomarker for types of cancer [18]. The significance role of ARGs in LUAD had not been fully clarified. Increasing evidences revealed that molecularly defined subtypes could provide novel strategies for the therapy and prognosis of lung cancer [4]. Thus, it is significant to develop effective prognostic signature and molecular subtype for lung cancer.

After obtained 27 differentially expressed aging-related genes (ARGs), we performed consensus clustering analysis, and three clusters of TCGA-LUAD patients with significant difference in prognosis, immune infiltration, chemotherapy ,and targeted therapy were identified. The result revealed that LUAD patients in cluster 3 had a poor overall survival. Moreover, further analysis suggested that LUAD patients in cluster 3 could be more resistant to commonly chemotherapy, targeted therapy, and immunotherapy. Increasing evidences revealed that molecular subtype classification of cancer with distinct biological characteristics could guide the development of precision treatment [19]. Ideal molecular subtype of LUAD was vital for the immune checkpoint blockade therapy and prognosis [20]. Our study identified three aging-related molecular subtypes of LUAD, providing more evidence for the precise treatment and prognosis improvement for LUAD.

Our study also identified 17 potential prognostic biomarkers for LUAD, including AGTR1, BUB1B, CAT, CDK1, CDKN2A, CDKN2B, EGR1, FOXM1, IL6, KL, LEPR, PPARG, PYCR1, RECQL4, SOCS2, TFAP2A, and UCHL1. Based on these prognostic biomarkers, we developed an aging-related prognostic signature using LASSO cox regression analysis. Interestingly, this prognostic signature had a better performance in predicting the 1-year, 3-year, and 5-year overall survival of LUAD. Previous studies had identified some prognostic signatures for LUAD. Lin et al. had developed pyroptosis-related prognostic signature in lung adenocarcinoma [21]. Another study also identified an immune-related signature that had a good performance in the prognosis of lung adenocarcinoma [22]. A robust ferroptosis-related signature could provide potential for the personalized outcome prediction for LUAD [23].

Actually, many aging-related signatures had been identified in cancers. In melanoma, a series of aging-related genes were associated with prognosis and responsiveness to immunotherapy [24]. Xue et al. also developed an aging-related prognostic signature for pancreatic adenocarcinoma [25]. Another study also constructed a prognostic signature based on 9-aging related genes for acute myeloid leukemia [26]. Moreover, another bioinformatics study also developed an aging-related signature, which had a good performance in risk stratification and prognosis prediction in lung squamous carcinoma [27].

Another vital finding of our study was that we also identified the lncRNA UCA1/miR-143-3p/CDK1 regulatory axis in LUAD. This regulatory axis may play a vital role of progression of LUAD. Previous study revealed that lncRNA UCA1 was a prognostic biomarker, and it could accelerate tumor proliferation and migration [28, 29]. Moreover, previous studies suggested that the miR-143-3p expression was downregulated in lung cancer and correlated with biological process regulation [30, 31]. CDK1 was upregulated in lung cancer, and it acts as a potential prognostic biomarker [32, 33]. Interestingly, the data of our study further verified these results. Further in vivo and in vitro studies should be performed to verify this regulatory axis.

## 5. Conclusion

Our study identified three clusters of TCGA-LUAD patients with significant difference in prognosis, immune infiltration, chemotherapy, and targeted therapy. We also developed an aging-related prognostic signature that had a good performance in the prognosis of LUAD.

## Data Availability

The analyzed data sets generated during the study are available from the corresponding author on reasonable request.

## Supplementary Materials

Supplementary Materials Supplementary Table 1: three hundred seven genes related to aging.
